# Transposable element expansion and low-level piRNA silencing in grasshoppers may cause genome gigantism

**DOI:** 10.1186/s12915-022-01441-w

**Published:** 2022-10-28

**Authors:** Xuanzeng Liu, Muhammad Majid, Hao Yuan, Huihui Chang, Lina Zhao, Yimeng Nie, Lang He, Xiaojing Liu, Xiaoting He, Yuan Huang

**Affiliations:** 1 College of Life Sciences, Shaanxi Normal University, Xi’an, China; 2 School of Basic Medical Sciences, Xi’an Medical University, Xi’an, China; 3 College of Life Science and Engineering, Henan University of Urban Construction, Pingdingshan, China

**Keywords:** Genome size, Transposable elements, TE transcripts, piRNA silencing, Grasshopper

## Abstract

**Background:**

Transposable elements (TEs) have been likened to parasites in the genome that reproduce and move ceaselessly in the host, continuously enlarging the host genome. However, the Piwi-interacting RNA (piRNA) pathway defends animal genomes against the harmful consequences of TE invasion by imposing small-RNA-mediated silencing. Here we compare the TE activity of two grasshopper species with different genome sizes in Acrididae (*Locusta migratoria manilensis*♀1C = 6.60 pg, *Angaracris rhodopa*♀1C = 16.36 pg) to ascertain the influence of piRNAs.

**Results:**

We discovered that repetitive sequences accounted for 74.56% of the genome in *A. rhodopa,* more than 56.83% in *L. migratoria*, and the large-genome grasshopper contained a higher TEs proportions. The comparative analysis revealed that 41 TEs (copy number > 500) were shared in both species. The two species exhibited distinct “landscapes” of TE divergence. The TEs outbreaks in the small-genome grasshopper occurred at more ancient times, while the large-genome grasshopper maintains active transposition events in the recent past. Evolutionary history studies on TEs suggest that TEs may be subject to different dynamics and resistances in these two species. We found that TE transcript abundance was higher in the large-genome grasshopper and the TE-derived piRNAs abundance was lower than in the small-genome grasshopper. In addition, we found that the piRNA methylase HENMT, which is underexpressed in the large-genome grasshopper, impedes the piRNA silencing to a lower level.

**Conclusions:**

Our study revealed that the abundance of piRNAs is lower in the gigantic genome grasshopper than in the small genome grasshopper. In addition, the key gene HENMT in the piRNA biogenesis pathway (Ping-Pong cycle) in the gigantic genome grasshopper is underexpressed. We hypothesize that low-level piRNA silencing unbalances the original positive correlation between TEs and piRNAs, and triggers TEs to proliferate out of control, which may be one of the reasons for the gigantism of grasshopper genomes.

**Supplementary Information:**

The online version contains supplementary material available at 10.1186/s12915-022-01441-w.

## Background

Metazoan haploid nuclear genome sizes (C-values) range from 0.02 to 132.83 pg and exhibit more than 6600-fold variation [1, 2]. Perplexingly, the large variation in genome size occurs between morphologically similar species, and gigantic genomes emerge in relatively simple organisms [3]. In the tree of life, species with gigantic genomes (larger than 10 GB) only account for a tiny fraction, including lungfishes [4], salamanders [5, 6], deep-sea crustaceans [7, 8], and orthoptera insects [9, 10]. The evolutionary mechanism of gigantic genomes has always been a mystery that researchers are eager to unravel. Some researchers found that DNA loss rates in the gigantic genomes of salamanders are significantly lower than in other vertebrates [5], and extensive DNA loss exists in birds and mammals with smaller genomes [5, 11]. Other researchers believe that rapid increases in genome size occur mainly through whole-genome duplications (WGD) or bursts in the activity of transposable elements (TEs) [12–14]. In general, differential expansion, accumulation, and removal of TE sequences are major determinants of genome size variation [15–17].

TEs, as selfish DNA, have the ability to move around and replicate themselves within the genomes [18–20]. At the onset of invasion, TEs start as a single copy in the host genome. Using the host's replication machinery, TEs rapidly expand the number of copies in each successive generation of the entire population [21]. TEs consume resources by hijacking cellular machinery to produce mRNAs and proteins necessary for transposition, and TEs expansion can directly disrupt genes or promoter regions [22, 23]. Even transposition-inactive TEs (“sleeping TEs”) can serve as substrates for ectopic recombination, along with other related TE insertions scattered throughout the genome [24–26]. Although some beneficial TE insertions have been found, such as conferring resistance to insecticides, at least a large fraction of the genome size variation caused by TE expansion can be attributed to non-adaptive processes [27, 28]. In the long-term struggle between TEs and the host genome, TEs have chosen the germline as the main battleground, where transposition events directly affect genome size variation and even the most deleterious TE insertions are heritable [29, 30]. In metazoans, the discovery of a small RNA-based defense system revealed that a genomic immune system restricts their selfish expansion by identifying active TEs [31, 32].

The Piwi-interacting RNA (piRNA) pathway is a critical regulator of germline TE activity. This host defense system relies on piRNAs that bind to PIWI-clade proteins and suppress TE activity transcriptionally and post-transcriptionally [33–36]. In *Drosophila melanogaster*, post-transcriptional silencing of TEs is based on Aubergine (AUB) and Argonaute3 (AGO3) binding directly to piRNAs, guided by the specific binding of piRNAs to TE transcripts [37–44]. Antisense piRNAs are thought to derive exclusively from TE-rich loci called piRNA clusters [33, 45]. The piRNA clusters are transcribed into multiple long precursor transcripts which are then cut and processed into small RNAs that are reverse complementary to TE transcripts [46, 47]. Additionally, the TE transcript is degraded through a “secondary” piRNA pathway to form sense piRNAs, which in turn produce more antisense piRNAs through the exact targeting and cleavage of antisense piRNA precursors, and this process is known as the “Ping-Pong cycle” [30, 33, 48, 49].

TE activity is highly dynamic during evolution, and the host genome faces a constant onslaught of reactivated or horizontally transferred TE families [50]. The hopping frequency and randomization of insertion sites allow TEs to exhibit strong sequence diversity [45, 51, 52]. The high dynamics and diversity of TEs allow some elements to escape the control of piRNAs during proliferation. Of course, the piRNA clusters also showed to be highly dynamic responses to TEs variation [53]. Therefore, it is challenging and exciting work to study the relationship between TEs expansion and piRNAs within and between species.

Orthoptera is the only known group of in the Insecta class with a significantly enlarged genome [10, 54] and the only group in invertebrates that includes species with genome size larger than 10 GB. Here, we selected two Acrididae (Orthoptera) species with different genome sizes (*Locusta migratoria manilensis*♀1C = 6.60 pg, *Angaracris rhodopa*♀1C = 16.36 pg) to investigate the genome repeat composition and evolutionary history of the TEs found in the two species using low-coverage Illumina sequencing short reads. The intraspecies comparison of the abundance of retrotransposon transcripts in different tissue types was also investigated using RNA sequencing data. Combined with the genomic content of retrotransposons, we compared the abundance of retrotransposon transcripts and piRNAs between the two species. Finally, we discuss the relationship between TEs and piRNAs within and between species. Our study provides new insights into the mystery of grasshopper genome gigantism.

## Results

### Exploration and comparison of repetitive sequences in two Acrididae genomes

**Fig. 1:**
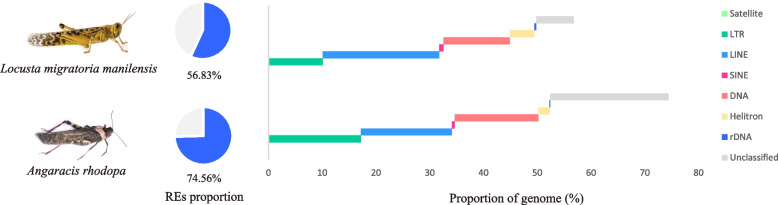
Content and composition of repetitive sequences in two genomes. The pie chart depicts the fraction of genome-wide repetitive elements. Bar graphs represent the proportion of repeat elements in the genome for each family. DNA: Class II DNA transposons; Unclassified: conflicting evidences and no evidence repeat

We used 0.1x genome coverage sequencing data to analyze the repeat content of the two species through the dnaPipeTE pipeline (see the “Methods” section). The results show that repetitive elements in *A. rhodopa* (16.36pg) account for 74.56% of the genome and 56.83% in *L. migratoria* (6.60pg) (Fig. 1). We present the genome size measurements in Additional file 1: Fig. S1. Long terminal repeats (LTRs), long interspersed nuclear elements (LINEs), and DNA transposons in the TE subclass account for most of the repetitive sequence content. The difference in LTR elements between the two species is significant; those in *A. rhodopa* account for 17.21% of the genome, while those in the *L. migratoria* only comprise 10.06% (Additional file 2: Table S1). When comparing the total content of TEs in the two species, TEs accounted for 52.28% of the *A. rhodopa* genome and more than 49.47% in the *L. migratoria* genome. In addition, the proportion of unclassified repetitive elements is also vastly different in *A. rhodopa* and *L. migratoria*, accounting for 22% and 7.01% of the genome, respectively. Since dnaPipeTE uses Repbase to annotate the found repeats, the database contains the repeats of *L. migratoria* but not *A. rodopha*, which makes the annotation results more friendly to *L. migatoria* and shows fewer unknown repeats. The detailed genome proportion of each repetitive element, including simple repeats and rRNA, is shown in Additional file 2: Table S1.

Within the TE subclasses, the proportions of DNA transposons, short interspersed nuclear elements (SINEs), and LTRs in the large-genome grasshopper are higher than those in the small-genome grasshopper (Fig. 1). However, it is surprising that the proportion of LINEs in the small-genome grasshopper is 21.72% higher than that in the large-genome grasshopper (16.87%). There are two possible reasons for this, one is that repeats homology-based annotation produces a more friendly bias towards *L. migatoria*, and the other is that LINEs proliferated more slowly than other TE subclasses in the process of increasing grasshopper genome size.

### Comparison of TEs expansion and evolutionary history in two species

**Fig. 2:**
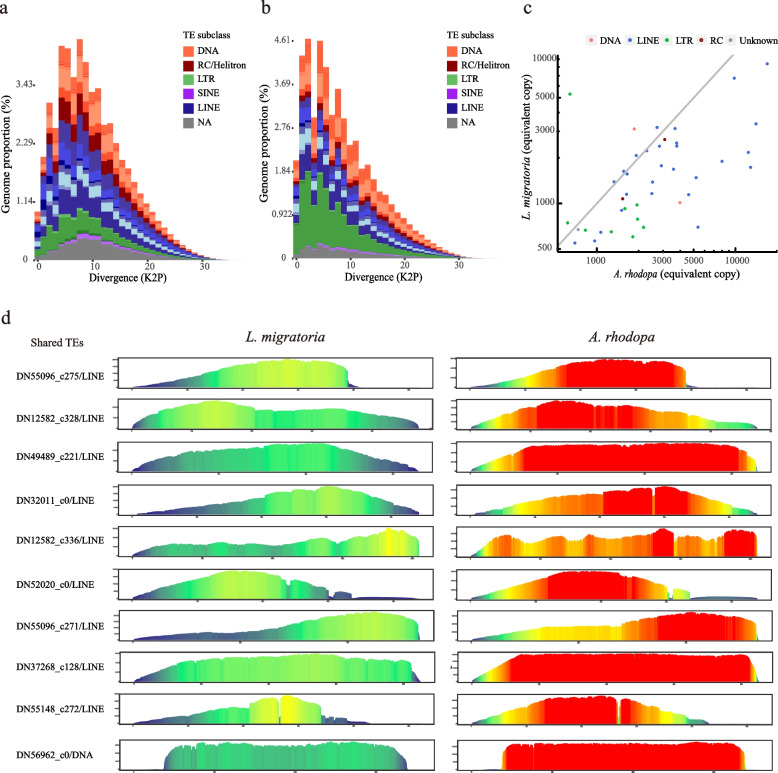
TE divergence landscapes and scaled profiles. **a** *L. migratoria* TEs landscape. The *x*-axis shows the level of divergence (Kimura 2-parameter distance) between each identified TE copy. The *y*-axis shows the proportion of the genome occupied by each bin. NA: Some TE annotation results not recognized by the script when drawing the landscape. **b** *A. rhodopa* TEs landscape. **c** Comparison of copy numbers of TEs shared by two species (copy number > 500). A dot represents a shared TE. **d** Repeat profiles of top 10 shared TEs present in two genomes. The *x*-axis represents the loci of the consensus sequence, and the *y*-axis is the depth of coverage for each position. The deeper the red color of the profile is the higher the coverage of reads. The repeat profiles of the remaining 31 shared TEs are shown in Fig. S2

To test whether the observed abundance patterns of specific TEs were driven by ancient proliferation events or by recent activities, we first generated divergence “landscapes” for TEs within each genome using dnaPipeTE (see the “Methods” section). The landscapes measure the amount of sequence divergence between each copy of TE. Histograms of the resulting Kimura 2-parameter distance (K2P) provide insights into the evolutionary history of TE activity [55–57]. The repeat landscape plot showed that the two species exhibit different patterns (Fig. 2a, b). From the landscapes of each TE subclass, the greatest difference between the two species is LTR, with the landscape peaking closer to the *y*-axis for *A. rhodopa* than for *L. migratoria* (Additional file 1: Fig. S2a)*.* In *A. rhodopa*, a large number of TE copies have very low divergence from the consensus sequence (K2P<5%). TE copies in *L. migratoria* deviate significantly from that of the consensus sequence (K2P = 5–10%). We discovered that *A. rhodopa* has a higher genome abundance than *L. migratoria* when comparing the least divergent (divergence < 1%) TEs elements from their consensus sequences*.* The results suggest that *A. rhodopa* has more newly proliferated TE copies, with little divergence from the consensus sequence. Assuming a molecular clock for nucleotide substitutions within duplicated TEs, a smaller divergence from the consensus sequence indicates a recent active transposition event [58]. We consider that the TEs have recently been actively transposed in *A.rhodopa*, while more ancient proliferation events occurred in *L. migratoria*.

TEs are extremely unconserved within and between species, and variation exists even between TE copies. In the TE comparative analysis of two species, it is crucial to find the consensus sequence shared between the two species. For this, we used the comparative analysis script of dnaPipeTE (see the “Methods” section), and the results revealed 162 shared repeats (copy number > 100) in the two species (Additional file 2: Table S2), with 41 TE sequences over 500 copies (Fig. 2c and Additional file 2: Table S3). We performed subsequent analyses with these 41 shared TEs (containing 9 LTRs, 28 LINEs, 2 DNA transposons, and 2 Helitrons). Upon comparing the copy numbers of shared TEs in the genomes of the two species, we observed that there are more TEs below the gray line (Fig. 2c), which indicates that most of the shared TEs accumulate higher copies in *A.rhodopa*.

Sequence variation and accumulation of repeat copies appeared in the proliferation process. The RepeatProfiler tool was used to analyze shared TEs to better observe the variation of repeat units between species (see the “Methods” section). The top 10 TEs with the highest number of copies are shown in Fig. 2d, and the scaled profile of the remaining 31 shared TEs is shown in Additional file 1: Fig. S3. The consensus sequence of the shared TEs we found exists in both species. The TE scaled profiles show the difference in the accumulation of TE copies, and overall the depth of reads coverage in *L. migratoria* is lower than in *A. rhodopa* (Fig. 2d and Additional file 1: Fig. S3). Furthermore, the profiles lend insight into repeat features. We discovered that the 5′ and 3′ ends were extended during TE class I proliferation whereas DNA transposons did not, and the extension from the origin to the 3′ and 5′ ends of LTR is not asymmetric.

### Transposition activity of TEs

In the TE divergence landscapes, we inferred that the sequences with K2P deviation < 5% have recently undergone frequent transposition events. In addition, the transposons transcriptome results can better illustrate the transcriptional activity of TEs.

**Fig. 3:**
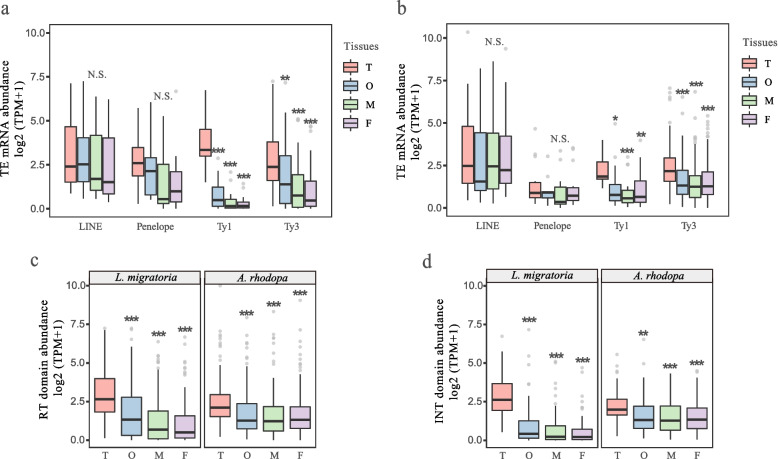
Transposition activity of TEs. The transcript abundance was normalized by TPM, and piRNA abundance was normalized by RPM. **a** TE transcripts abundance differences in *L. migratoria*. **b** TE transcripts abundance differences in *A. rhodopa*. **c** RT domain transcripts abundance in two species of different tissues. **d** INT domain transcripts abundance in two species of different tissues. Abbreviations are defined as T = testis; O = ovary; M = male body; F = female body. Significance is denoted by * *p*<0.05; **p<0.01; *p*<0.001; NS *p*>0.05 (method = *T*-test)

We performed TEs transcriptional expression analysis using RNA-seq data of four tissue types (testis, ovary, male body, female body) in both species (the body sample is a mix of head, thorax, and leg). We obtained sequencing data for three biological replicates per tissue sample. After assembly, we used the DANTE tool and extracted the domains of group-specific antigen (GAG), protease (PROT), reverse transcriptase (RT), ribonuclease H (RH), and integrase (INT) for retrotransposon structural annotation (see Methods). Finally, we discovered 101 retrotransposon transcripts (TPM>1) in L. migratoria and 154 transcripts (TPM>1) in *A. rhodopa* from four subclasses of retrotransposon transcripts (LINE, Penelope, LTR/Ty1 copia, LTR/Ty3 gypsy) (Additional file 2: Table S4–S6). Different families of retrotransposons exhibit different tissue specificities. In the analysis of retrotransposon transcript abundance in different tissues, we found that the two species exhibited similar patterns, with significantly high expression of LTR/Ty1_copia and LTR/Ty3_gypsy in testis (*T*-test). In contrast, LINE and Penelope elements did not display tissue-specific expression (Fig. 3a, b).

Transcription of retrotransposons is only the first step in the entire transposition event, which is followed by reverse transcription and integration. We also investigated the expression differences of reverse transcriptase and integrase in tissues. Analysis of transcript abundance of the RT and INT domains revealed that reverse transcriptase and integrase were highly expressed in testis tissue (Fig. 3c, d).

**Fig. 4:**
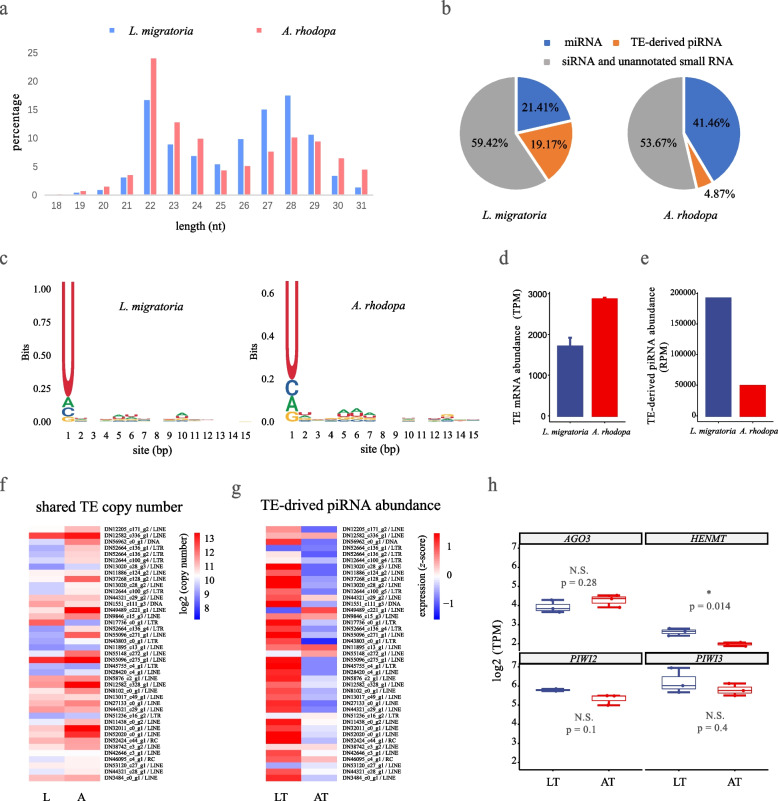
piRNA silencing mechanisms between two species. **a** Length distribution of small RNAs in two species. **b** Proportion of small RNA in the two species. **c** Base bias visualization of TE-derived piRNAs. **d** Total abundance of TE transcripts in testis. **e** Total abundance of TE-derived piRNAs in testis. **f** Heatmap of 41 shared TE copy number. The value of copy number is shown in Additional file 2: Table S3. **g** Heatmap of TE-derived piRNAs abundance. piRNA abundance was normalized by RPM, and the heatmap was plotted using log2(RPM) and scale = “row” parameters. **h** Analysis of differential expression of four genes in the piRNA pathway. The *y*-axis represents log2 transcript abundance (TPM). Abbreviations are defined as L, *L. manilensis*; A, *A. rhodopa*; LT, *L. migratoria* testis; AT, *A. rhodopa* testis. Statistical differences are represented as * *p*<0.05; ** *p*<0.01; *** *p*<0.001; N.S. *p*>0.05 (method = *T*-test)

Comparing the retrotransposon activity of two species can be problematic. The retrotransposon transcripts in the two species do not correspond and have little in common. Therefore we compared the total abundance of retrotransposon transcripts in the testis tissue of the two species. The total abundance of retrotransposon transcripts was higher in the large-genome size species *A. rhodopa* (Fig. 4d). However, TE transcriptional activity and post-transcriptional silencing jointly determine transcript abundance. We therefore analyzed the effect of piRNAs on post-transcriptional silencing of TEs.

### Comparison of piRNA silencing level between two species

We performed small RNA sequencing on testis tissue from both species. First, rRNA, tRNA, and snRNA were removed from small RNA sequencing data (see Methods). Reads of mapped rRNA accounted for 4.90% (*L. migratoria* testis) and 10.13% (*A. rhodopa* testis) of clean data, respectively. After that, we performed length statistics on the remaining small RNAs. The small RNAs in the testis of the two species showed different length distributions (Fig. 4a). *L. migratoria* has a higher proportion of small RNAs with lengths of 27–28 nt. However, in *A. rhodopa* small RNAs with a length of 22 nt are in the majority. The length of piRNAs is about 23–30 nt [59], so we speculate that there may be more piRNAs in *L. migratoria*. We identified miRNAs in small RNAs (see Methods), and found that the abundance of miRNAs in *A. rhodopa* was higher than that in *L. migratoria*. The *A. rhodopa* small RNAs consisted of 41.46% of miRNAs. Meanwhile, miRNAs in *L. migratoria* only accounted for 21.41% of total small RNAs. The remaining small RNAs were aligned with TEs to identify TE-derived piRNAs (see the “Methods” section). We selected aligned small RNAs with a length of 23–31 nt and identified them as TE-derived piRNAs. We performed base bias analysis on the identified TE-derived piRNAs. 82.3% and 72.3% of the TE-derived piRNAs in *L. manilensis* and *A. rhodopa*, respectively, had uridine in the first position of the 5′-end (referred as “1U”) (Fig. 4c). TE-derived piRNAs accounted for 19.17% of all small RNAs in *L. migratoria*, while piRNAs in *A. rhodopa* only accounted for 4.87% of all small RNAs.

There was a clear difference in the total abundance of TE-derived piRNAs in the two species. The small-genome grasshopper has a higher TE-derived piRNAs abundance of 191,701.54 (reads per million; RPM), while the large-genome grasshopper has a lower TE-derived piRNAs abundance of 48,702.81 (RPM) (Fig. 4e). We found that the small-genome species with low abundance of total TE-derived piRNAs corresponded to a higher abundance of transposon transcripts (Fig. 4d). We suspect that piRNA silencing was more effective in species with smaller genomes than in species with larger genomes. In addition, we discovered that the TE transcriptional expression analysis for the ovary was consistent with the testis (Additional file 1: Fig. S4a, b).

Identification of the TEs shared by the two species could serve as a bridge to explore the effects of piRNA silencing on TE accumulation across species. First, the heatmap and scatterplot showed the copy numbers of 41 shared TEs in both species, with 34 of the 41 TEs accumulating more copies in *A. rhodopa* (Figs. 2c and 4f). Second, we compared the piRNAs corresponding to each shared TE in the two species. The result shows that the abundance of piRNAs corresponding to TEs was lower in the large-genome grasshopper (Fig. 4g and Additional file 2: Table S7). Overall, we suggest that the large-genome grasshopper with a low piRNA abundance is more susceptible to TE invasion.

To explore what causes the low abundance of piRNAs in the large-genome grasshopper, we analyzed key genes in the piRNA pathway, including AGO3, PIWI2, PIWI3 (homologous to *Drosophila* AUB), and HEN methyltransferase 1 (HENMT) in the gonads [60–63]. The Piwi protein family did not display significant differences between the two species in the testis (Fig. 4h). Notably, HENMT was significantly different in the testis between the two species. HENMT protects the 3′-end of piRNAs from uridylation activity and subsequent degradation, by acting as a methyltransferase that adds a 2′-O-methyl group at the 3′-end of piRNAs [64, 65]. We reasoned that the low expression of HENMT in the large-genome grasshopper resulted in piRNA silencing at a low level.

We found similar results in the ovary (Additional file 1: Fig. S4b, c), evidence that the low expression of HENMT in large-genome grasshoppers impairs the piRNA silencing mechanism. Although some studies suggest that AGO3, PIWI, and AUB play an important role in the repression of TE transposition [37, 59], we have no evidence that these genes are significantly different in the two grasshopper species (Fig. 4h).

### Correlation analysis between piRNAs and TE transcripts

The Ping-Pong cycle is a keystone in the piRNA pathway, which allows antisense piRNAs to silence more TE transcripts to generate more sense piRNAs and increase the overall abundance of piRNAs. We speculated a positive correlation between TE transcripts and TE-derived piRNAs.

**Fig. 5:**
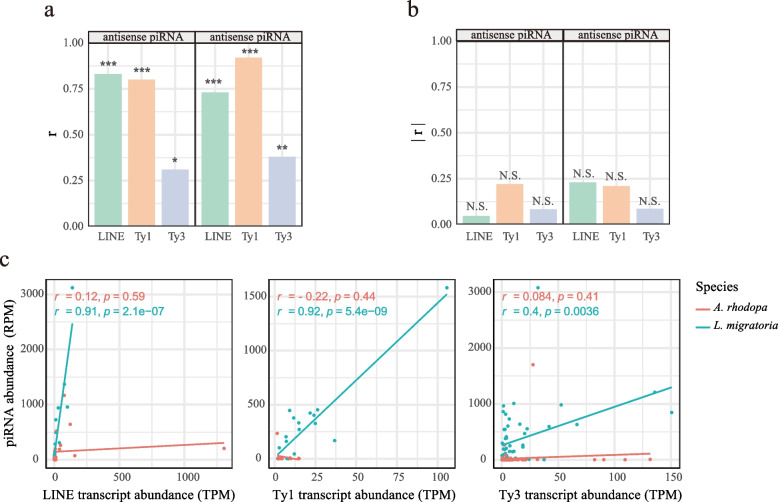
Correlations between TE mRNA abundance and TE-derived piRNA abundance. **a** Correlation analysis of sense and antisense piRNA with TE transcript abundance in *L. migratoria*. **b** Correlation analysis of sense and antisense piRNA with TE transcript abundance in *A. rhodopa*. **c** Linear fitting of piRNA abundance and transcript abundance of TEs. r represents the Pearson correlation coefficient, with statistical significance noted as * *p*<0.05; ** *p*<0.01; *** *p*<0.001; N.S. *p*>0.05

To test this hypothesis, we evaluated the relationship between the abundance of retrotransposon transcripts and the abundance of corresponding sense and antisense piRNAs in the testis. The piRNA abundance corresponding to retrotransposon transcripts is shown in Additional file 2: Table S8–S11. In *L. migratoria*, both sense and antisense piRNAs of LINE and Ty1_copia elements showed significantly strong correlations with transcript abundance (antisense LINE: *r*=0.83 p=2.2e−05; antisense Ty1: *r* = 0.8, p=1.1e−05; sense LINE *r*= 0.73 *p*= 0.00059; sense Ty1: *r*=0.92, *p*= 2.5e−09; Pearson correlation coefficient), and Ty3_gypsy elements showed relatively weak correlations (antisense: *r*= 0.31 *p*= 0.026; sense: *r*=0.38, *p*= 0.0058; Pearson correlation coefficient) (Fig. 5a). These *L. migratoria* results confirmed our hypothesis that TE-derived sense and antisense piRNAs abundance positively correlate with TE transcripts abundance. We did not perform a correlation analysis for Penelope elements because of the few annotated entries by Penelope transcripts.

In contrast, there was no significant correlation between sense and antisense piRNA abundance with the abundance of retrotransposon transcripts in *A. rhodopa* (LINE, Ty1_copia, Ty3_gypsy) (Fig. 5b). We suspect that this uncorrelation is due to impaired piRNA silencing machinery in *A. rhodopa*. Moreover, the impaired piRNA pathway simultaneously affects sense and antisense piRNAs (Fig. 5b).

We additionally compared the relationship between piRNAs and TE transcripts abundance for the two species. We chose to display the total piRNA abundance as this is consistent with the analysis of both sense and antisense piRNA abundance (Fig. 5c). Linear fittings were performed within species and we discovered a significant positive correlation in *L. migratoria* but no correlation in *A. rhodopa*. We also found that the slope of the fitted line was always greater for *L. migratoria* than for *A. rhodopa* (Fig. 5c). As the fitted line leans more towards the *x*-axis, the slope is closer to 0, indicating that the abundance of piRNA is low in the *L. migratoria* species, while the abundance of TE transcripts dominates. Fitted lines leaning toward the *y*-axis indicate species with better piRNA silencing and TE transcripts at lower abundance. In the large-genome *A. rhodopa*, the fitted line is closer to the *x*-axis, and the slope of the fitted line for the Ty1_copia element is smaller than 0 (Fig. 5c), which may be related to the rapid expansion of some TEs. Furthermore, we performed the same analysis on the ovaries of both species. We found that the relationship between TE transcripts with TE-derived piRNAs was consistent in the testis and ovary (Additional file 1: Fig. S4d).

## Discussion

### Comparison of repetitive sequences and highly dynamic TEs

Exploration of repetitive sequences using unassembled raw genome data is at a loss compared to the full assembled genome. Previously, the repeatome analysis of *L. migratoria* using assembled genome data discovered 58.86% of the repetitive sequences in the whole genome [66, 67], while our results were slightly smaller at 56.83%. Of course, the complete assembled genome is preferable for comparing the repeat sequences of two species. However, for large-genome species lacking an assembled genome, low-coverage reads can be used for repeat sequence comparison. By using sequencing data with 0.1× genome coverage in the large-genome grasshopper, we found that repetitive sequences accounted for 74.56% of the genome. A recent study of more than 600 insects using assembled genome data analysis revealed the proportion of repeats in insect genomes ranged widely from 1.6 to 81.5% [68]. If giant genome grasshopper species have assembled genome data in the future, a higher proportion of repetitive sequences may be found.

The proportion of repetitive sequences in the genome is a relative value. We found that the proportion of rDNA and LINE in the small-genome grasshopper species is higher than that in large-genome grasshopper species. This does not mean that rDNA and LINE are not replicated during the genome expansion, but rather replication is relatively slow compared to other repetitive elements. From the multiples of repeats accumulated, the total length of LTR in *A. rhodopa* increased by 4.24-fold compared with that in *L. migratoria*. We believe that LTRs contributed the most to the grasshopper genome size variation. Here we need to be clear that there may be a more friendly bias towards *L. migratoria* when repeating homology annotations, since the reference database used contains repeat entries for *L. migratoria*. This bias may have an impact on the ratio of annotated repeats to unknown repeats, but not on the total repeats content in the species’ genome.

Repeated regions generally evolve much more rapidly than single-copy DNA sequences. Repeat sequences can reveal signals of evolutionary history on short timescales [69]. We found that TEs are highly dynamic both within and between species. Both *L. migratoria* and *A. rhodopa* belong to the Oedipodinae subfamily, but most of the TEs are unique to each other. We are not surprised by this result, as few identical TEs were inserted in the more closely related *D. melanogaster* and *D. simulans* [70, 71].

### TE expansion patterns in genome size evolution

Insect genomes are characterized by high heterozygosity and duplication [72], and genome size variation is an extremely complex process. The grasshopper species *Bryodemella holdereri* has the largest genome size identified to date among insects (1C value = 18.64 pg) [9], which is approximately 260-fold larger than the smallest insect genome (*Clunio tsushimensis*, 1C value =0.07 pg) [73–75]. Thus, genome sizes vary greatly among insects. Consistent with the C-value paradox [76], studies in the orders Strepsiptera, Hymenoptera, and Dictyoptera found that genome size was not phylogenetically related to the inherent traits of these insects [77–79].

Our study of the TEs divergence landscape found that TEs exhibited distinct burst patterns between species (Fig. 2a, b). The large-scale outbreak of TEs in the small-genome grasshopper occurred in a more ancient period, and recently the TEs have ended the outbreak without rapidly accumulating copies. However, TEs in large-genome grasshoppers are still in an active stage of rapid accumulation. The period of rapid TE accumulation in the species is not homogenized. LINE and DNA transposons also showed different burst patterns in the diverse insect order Trichoptera [80]. TEs in fish genomes also have distinct accumulation patterns [56]. Our results found that LINE and SINE transposons have different burst periods (Additional file 1: Fig. S2b, c). The non-synchronous burst and expansion of TEs may be one of the reasons why genome size variation has no phylogenetic signal.

To compare differences in TE copy accumulation across species, we performed a scaled-profiles analysis for shared TEs. On a copy number scale, the giant genome grasshopper species has a higher copy number of repetitive elements. It is assumed that these shared-TEs existed during the common ancestor of the two species, and these TEs have undergone the same temporal evolution in different hosts. We observed that these TEs accumulate more copies with a faster expansion rate in *A. rhodopa*. In scaled profiles, we consider the position with the highest read coverage as the TE origin, which is accompanied by an increase in copy number and extension of the sequence end as TE jumps and proliferates. We found that the large-genome grasshopper has more copies of end extensions.

In addition, the TE divergence landscapes showed that active transposition events have recently occurred in *A. rhodopa*, whereas degeneration or inactivation of TEs has occurred in *L. migratoria*. The different landscape patterns in the two species illustrate that TEs are subject to different dynamics and resistances as they expand. TEs suffer different fates, which may be related to the host defense mechanism against TE invasion.

### TE activity in different tissues

TEs choose the main battlefield in the germline, where even the most harmful TE insertions are heritable [81, 82]. Somatic transposition is considered a dead-end in TE evolution, with no long-term effects on the host but a more selective burden on the self-reproduction of TEs [83]. We found that TE has higher transcriptional activity in the testis, and this difference in TE activity between different tissues is consistent in *L. migratoria* and *A. rhodopa*. Similar results were found in a study of *D. melanogaster* and *D. simulans* [84].

The transposition of TE in somatic cells cannot be ignored. Although its transposition in somatic cells will not be inherited, it will harm the adaptability of the host. In humans, the expression and transposition of LINE elements have been detected in various somatic contexts, including early embryos and certain stem cells [85, 86]. Somatic activity has also been observed in human cancers, where tumors can acquire hundreds of new LINE-1 insertions [87–89]. Our results did not find significant differences in LINE between tissues, which may indicate that LINE elements are also highly expressed in body tissues.

### Effects of piRNA silencing on TE activity

We compared the abundance of piRNAs in the testis and ovary of the two species. After all, the transposition event in the germline directly affects the genome size variation of the species. Before discussing the effect of piRNA silencing on TE activity, we teased out the relationship between TE age, activity, and abundance. We calculated the K2P divergence of 41 shared TEs in the two species genomes using RepeatMasker (Additional file 2) (see Methods). The K2P distance from the consensus sequence reflects recent TE activity and the time since the insertion of a TE copy [20, 55, 58]. Within species, we found no significant correlation between K2P distance and the abundance of TEs (*L. migratoria*: *r* = −0.13, *p* = 0.42; *A. rhodopa*: *r* = −0.18, *p* = 0.27; Pearson correlation coefficient) (Additional file 1: Fig. S5a). In addition, there was no significant correlation between K2P distance and piRNA abundance in *A. rhodopa* (*r* = -0.29, *p* = 0.062), but there was a negative linear correlation in *L. migratoria* (*r* = −0.46, *p* = 0.0026) (Additional file 1: Fig. S5b). This is consistent with the analysis of transcriptome, the more active TE in *L. migratoria* corresponds to the higher abundance of piRNA.

Two points need to be clarified when comparing piRNA silencing levels across species. First, piRNAs can silence multiple transposons with reverse complementary sequences. Second, a transposon can hold a transposase encoded in other transposons to complete its transposition [90, 91]. The relationship between TEs and piRNAs is not a one-to-one correspondence, so we analyzed the total abundance and the abundance of each TEs separately. This inter-species difference was consistent in total abundance and abundance per TE, with high piRNA abundance in the small-genome grasshopper and low piRNA abundance in the large-genome grasshopper. The low abundance piRNA pool has resulted in the large-genome grasshopper exhibiting higher TE transcripts abundance.

The “trap model” holds that an invading TE proliferates within the host until at least one copy jumps into a piRNA cluster (trap), which triggers the production of piRNAs [92–94]. Based on this model, we believe that the higher the TE activity in the genome, the higher the probability of TEs jumping into the piRNAs cluster, and the more piRNAs will be generated. However, the low piRNA abundance exhibited in the large-genome grasshopper contradicts this model. When we compared the expression of key genes in the piRNA pathway between the two species, we found a differentially expressed gene, HENMT. It is directly related to piRNA abundance and protects the 3′-end of piRNAs from degradation. Research on germ cells in adult male mice showed that loss of HENMT function and the concomitant loss of piRNAs resulted in TE derepression in adult meiotic and haploid germ cells [65]. The low expression of HENMT causes piRNAs to be more easily degraded, which may explain why the abundance of piRNAs in the large-genome grasshopper is lower than that in the small-genome grasshopper.

The germline-specific ping-pong amplification cycle has been demonstrated to produce antisense piRNAs from piRNA precursor transcripts, and the TE transcript is degraded to form sense piRNAs through a “secondary” piRNA pathway [30, 95]. More antisense piRNAs can be generated by precise targeting and cleavage of antisense piRNA precursors by sense piRNAs. The ping-pong cycle is a keystone of the piRNA pathway because it both silences TEs post-transcriptionally and enhances the silencing capacity of the pathway by producing more piRNA [95, 96]. We believe that low expression of HENMT causes impairment of the piRNA silencing mechanism in the large-genome grasshopper. As the ping-pong amplification cycle amplifies, this effect results in piRNA silencing at a lower level.

### Low-level piRNA silencing may disrupt the balance between TEs and piRNAs

It is interesting to discuss the relationship between piRNAs and TEs within species because piRNAs originate from TE-rich regions of the genome and can inhibit TE transposition. A study of the Global Diversity Lines (GDL) of *D. melanogaster* revealed the existence of an evolutionary arms race between the copy numbers of TEs and the abundance of piRNAs [97]. In addition, another study also pointed out that TE mRNA abundance was positively correlated with TE-derived piRNA abundance [30]. These results validate a hypothesis that piRNA abundance correlates with the transpositional activity of a TE family, with the most recently active TE families being the most abundant among TE-derived piRNAs [30, 98].

Our findings in the grasshopper species with the smaller genome are consistent with the above hypothesis, but not in the grasshopper with the larger genome. We speculate that the uncorrelated association between TE mRNA abundance and TE-derived piRNA abundance in large-genome grasshoppers is due to weaker suppression of particular TE transcripts by low-abundance piRNAs, which leads to rapid accumulation of these TEs. The slope of the *L. migratoria* fitted line is always greater than that of *A. rhodopa*, suggesting that piRNAs have better control over TE transcripts in the small-genome grasshopper. For example, if TE transcripts have the same abundance in both species, the abundance of corresponding piRNAs is higher in *L. migratoria* than that in *A. rhodopa*.

The positive correlation between TEs and TE-derived piRNAs found in *Drosophila* and *L. migratoria* is considered a balanced relationship for the host to counteract the damage suffered by TE invasion under normal conditions. The low-level piRNA silencing in the large-genome grasshopper species disrupts the original balance between TEs and piRNAs, causing some TEs to be out of control and continue to expand.

### The adaptive cost of TE in the gigantic genome grasshopper

Natural selection and genetic drift are powerful forces shaping the distribution and accumulation of TEs [83]. Some studies suggest that piRNAs can significantly reduce the adaptive cost of TEs, and TE insertions that generate piRNAs are favored by natural selection [99–101]. Furthermore, some studies have shown that many protein components of the piRNA pathway show signatures of adaptive evolution [102–105]. From the host adaptation, the low-level piRNA silencing in the gigantic genome grasshopper species appears to be disadvantageous. The piRNA pathway is considered an adaptive defense in the transposon arms race [31]. If piRNAs fail to counteract the harm of TEs in giant genome grasshoppers, is there another mechanism to offset the adaptive cost of TEs.

There may be another possibility that the expansion of TEs may bring some evolutionary advantages to the host. Large amounts of DNA insertion or deletion would result in a high genome plasticity [106]. Research has shown that the proliferation of DNA transposons and LINEs in deep-sea species might play an important role in shaping highly plastic genomes and helping them adapt to the deep-sea environment [8]. We speculate that the expansion of TEs in the giant genome grasshopper species might help them better adapt to the living environment, because the *A. rhodopa* species was collected at higher altitude areas (average altitude of 3000 m). At present, we do not have enough evidence to prove this conjecture, and we need more samples from extreme living environments. Many questions about the host’s response to TE invasion remain unanswered. Whether this low-level piRNA silencing is unique to gigantic genome grasshopper species, or is an evolutionary process of Acrididae insects, requires more species data to reveal.

## Conclusions

In this study, we analyzed the activity of TEs in two grasshopper species using genomic and transcriptomic datasets. The results showed that the transposition of TEs was more active in *A. rhodopa*, which has a larger genome (16.36 pg). We found that the expansion of TEs in the large-genome grasshopper species was more rapidly manifested by the accumulation of more repeat copies. The different levels of the two hosts in response to TE invasion may be the main reason for the different expansion patterns of TEs in the two grasshopper species. By comparing the piRNA silencing mechanisms of the two species, we found that the piRNA methylase HENMT, which is underexpressed in the large-genome grasshopper, made piRNA abundance lower than that in the small-genome grasshopper, breaking the original balance between TEs and piRNAs. In summary, we hypothesize that low levels of piRNA silencing lead to an imbalance in the relationship between TEs and piRNAs in the host, resulting in a rapid expansion of TEs leading to genomic gigantism.

## Methods

### Materials and sequencing

Samples of *L. migratoria* under experimental rearing conditions and *A. rhodopa* were collected at the Tibetan Autonomous Prefecture of Haibei, Qinghai, China (36°52′47.45″N, 100°52′35.1″E) in August 2020. Live adults were taken to the laboratory for dissection. The DNA-grade samples were added to 95% ethanol and stored in a −20°C freezer. The RNA-grade tissues from males (head, leg, thorax, and testes) and females (head, leg, thorax, and ovary) were dissected and stored in RNAlater (Thermo Fisher Scientific, Waltham, USA) stored at −80°C until subsequent RNA extraction. The head, thorax, and legs of individual genders were mixed into one sample as a body tissue for RNA extraction. We sampled three biological replicates for each tissue sample. Furthermore, the freshly collected samples were used to estimate the genome size using flow cytometry (FCM) of propidium iodide-stained nuclei following the standard protocol [107, 108].

We extracted the genomic DNA of *A. rhodopa* from the hind leg of one female using an SDS-based lysis method and purified the DNA with chloroform. The extracted DNA was sonicated to a fragment size of 350 bp. The library was fixed onto a microarray by bridge PCR and sequenced using the Illumina HiSeq 2500 sequencing platform (PE150bp). The genomic data for *L. migratoria* was downloaded in the Sequence Read Archive (SRA), accession number SRR764584.

RNA sequencing libraries were generated using NEBNext Ultra RNA Library Prep Kit for Illumina (NEB, Ipswich, USA). The clustering of the index-coded samples was performed on a cBot Cluster Generation System using TruSeq PE Cluster Kit v3-cBot-HS (Illumina). The library preparations were sequenced on an Illumina NovaSeq 6000 platform, and paired-end reads were generated. Small RNA Sequencing libraries were generated using NEBNext Multiplex Small RNA Library Prep Set for Illumina (NEB). After that, the different libraries are pooled according to the effective concentration and the target amount of data off the machine, and 50 bp single-end reads are generated by Illumina NovaSeq 6000 sequencing.

### Exploration and comparison of repetitive sequences in two genomes

We used 0.1× genome coverage sequencing data for repeat sequences analysis with dnaPipeTE software [109]. The dnaPipeTE software installation and operation are as follows (sudo docker pull clemgoub/dnapipete:latest)( python3 dnaPipeTE.py -input Aread.fq -output -RM_lib Orthoptera.repeatmasker.lib -genome_size -genome_coverage 0.1 -sample_number 2 -RM_t 0.2 -contig_length 350). The -RM_lib parameter is the choice of the database, and there are two options to choose RepeatMasker Libraries (RepeatMasker.lib, a repository of protein sequences identified in transposable element) or construct a repeat sequence library ourselves. We selected a non-redundant database constructed from repeats of five Orthoptera species (with complete genome assembly), and the annotation results of this method outperformed RepeatMasker.lib. The Orthoptera.repeatmasker.lib is available in the figshare database (10.6084/m9.figshare.21256878).

TE landscapes are automatically generated in the dnaPipeTE output file. The consensus sequences used in the TE divergence landscape analysis are the respective annotated TEs in each species. We used dnaPT_compare.sh (https://github.com/clemgoub/dnaPT_utils) in dnaPipeTE to perform the comparative analysis of repeat sequences in the two species (dnaPT_compare.sh -A Locus_dnaPipeTE.OUT -a Locus -B Angar_dnaPipeTE.OUT -b Angar -o compare.out -T -e 500 -E -C 36). We selected shared TEs with more than 500 copies in both species for display.

### RepeatProfiler analysis of shared TEs

We used the RepeatProfiler tool (https://github.com/johnssproul/RepeatProfiler) for visualizing and comparing repetitive DNA profiles of 41 shared TEs from 0.5× coverage short-read sequence data [69],with the following command (repeatprof pre-corr -p data_folder; repeatprof profile -TE.fa data_folder -corr).

### Transcriptome assembly and TE transcripts annotation

Raw reads from all libraries were processed to remove sequencing adaptors and low-quality bases on the 3′ end using trimmomatic v0.39 [110], and clean reads were assembled using Trinity v2.9.1 [111] (Trinity --seqType fq --samples_file --SS_lib_type RF). We use Trinotate v3.1.1 (https://github.com/Trinotate/Trinotate.github.io/wiki) to annotate the assembly results. UniProtKB (https://www.uniprot.org/) and Pfam [112] reference databases were used for the analysis. Transcripts are compared with known protein databases through diamond (v0.9.14) [113] (diamond blastp --query --db --max-target-seqs 1 --outfmt 6 --evalue 1e-5) and hmmer (v3.3.1) (hmmscan --cpu -domtblout Pfam). We extracted key genes in the piRNA biogenesis pathway (Ping-Pong cycle) from the annotation results. The annotation of TE transcripts was done through Domain Based ANnotation of Transposable Elements (DANTE) (https://repeatexplorer-elixir.cerit-sc.cz/galaxy). We choose taxon and protein domain database version as REXdb (Metazoa_version_3.1). The minimum similarity parameter is set to 0.65, and the minimum alignment length parameter is set to 0.7. The structure of LTR retrotransposons and retroviruses are very similar, and they also encode a viral particle coat (GAG) and reverse transcriptase (RT), ribonuclease H (RH), and integrase (IN) [114]. According to the structure of Ty1_copia elements, it encodes the following protein domains (GAG-PROT-INT-RT-RH) and Ty3_gypsy elements encode (GAG-PROT-RT-RH-INT) protein domains [115, 116]. We annotated transcripts with three domains of INT, RT, and RH identified as LTR/copia and LTR/gypsy transcripts. We annotated the transcripts of LINE and Penelope according to their characteristic RT domains [117].

### Expression analysis of retrotransposon transcripts, transposase, and piRNA pathway key genes

Quantitative analysis of all transcripts was performed through the align_and_estimate_abundance.pl (https://github.com/trinityrnaseq/trinityrnaseq/tree/master/util) script (--transcripts --samples_file --est_method RSEM --aln_method bowtie2 --trinity_mode --prep_reference --thread_count). Then we extracted the TPM normalized expression matrices of retrotransposons and piRNA pathway genes separately based on DANTE, Pfam, and UniProtKB annotation results. Boxplots of each transcript abundance of retrotransposons and piRNA pathway genes were plotted by R packages (“ggplot” and “ggboxplot”), and significant differences were performed using *T*-test.

### TE-derived piRNAs identification

We deep-sequenced small RNAs from testis, ovaries, and bodies of *L. migratoria* and *A. rhodopa* individually. For these small RNA-Seq data, the 3′-adaptor sequences were removed using the Cutadapt (v3.3) [118] software and trimmed small RNA reads were 18–31 nt in length (cutadapt -a AGATCGGAAGAGCACACGTCTGAAC -m 18 -M 31). The processed reads were compared to the Rfam [119] database using bowtie (v1.3.0) [120] to remove rRNA, tRNA, and snRNA (bowtie -f -a --best --strata -p --al --un). Bowtie was then used to identify miRNAs, with the reference sequence as the miRNA hairpin sequence of *L. migratoria* [121–123]. The remaining small RNAs were mapped to TEs and TE transcripts, of which 23-31nt aligned reads were considered TE-derived piRNAs [97, 124] ( bowtie -v 3 -a reads.fa -S --al --un -f). Small RNA length statistics were generated through Shell command (grep -v '>' smallRNA.fa | perl -alne print length | sort |uniq -c). Graphical visualization of piRNA base bias was constructed by the R package “ggseqlogo” (R script: ggseqlogo (fasta_input, method="bits", seq_type="rna")).

### Correlation analysis and linear fitting of TE and piRNA

TE mRNA abundance and TE-derived piRNA abundance were normalized using TPM and RPM, respectively. An R script was used for linear fitting, supported by Pearson’s correlation test. (R script: ggplot (data, aes(x, y)) + geom_point ()+ geom_smooth (method = 'lm', formula = y ~ x, se = F) + stat_cor(data, method = "pearson")).

### TEs divergence analysis

We used RepeatMasker (http://repeatmasker.org) with the “-a” option and the RMBlast search engine to estimate the divergence of each shared-TEs (RepeatMasker 0.1x.fa -lib 41sharedTEs.fa -a -e rmblast) (calcDivergenceFromAlign.pl -s name.divsum name.fasta.align) (createSatellitome1Landscape.pl -div name.divsum -g genome_size).

## Supplementary Information

**Additional file 1: Fig. S1.** Flow cytometry estimation of the genome size for *A. rhodopa* female and male. **Fig. S2.** TE subclass landscapes of two species. **Fig. S3.** Repeat profiles of the remaining 31 shared TEs between two species. **Fig. S4.** Low-level piRNA silencing in *A.rhodopa* ovary. **Fig. S5.** Correlation analysis of K2P distance of TE with TE abundance and piRNA abundance.

**Additional file 2: Table S1.** Proportion of repetitive elements in the genome. **Table S2.** Comparative analysis of repeat sequences in two species. **Table S3.** List of 41 TEs shared in the two species (copy number >500). **Table S4.** Annotations of Class I retrotransposon transcript (TPM>1). **Table S5.** Retrotransposon transcript quantification matrix (TPM normalization) of *L. migratoria.* **Table S6.** Retrotransposon transcript quantification matrix (TPM normalization) of *A. rhodopa.* **Table S7.** Abundance of piRNAs corresponding to 41 shared TEs (RPM normalization). **Table S8.** Abundance of sense piRNAs corresponding to *L. migratoria* retrotransposon transcripts (RPM normalization). **Table S9.** Abundance of antisense piRNAs corresponding to *L. migratoria* retrotransposon transcripts (RPM normalization). **Table S10.** Abundance of sense piRNAs corresponding to *A. rhodopa* retrotransposon transcripts (RPM normalization). **Table S11.** Abundance of antisense piRNAs corresponding to *A. rhodopa* retrotransposon transcripts (RPM normalization). **Table S12.** List of K2P divergence and abundance of 41 shared-TEs.

## Data Availability

The *A. rhodopa* genomic sequencing data has been deposited at the public NCBI under SRA database SRR19352342 (https://www.ncbi.nlm.nih.gov/sra/SRR19352342) [125]. The RNA sequencing and small RNA sequencing data have been deposited at the public NCBI under Bioproject ID PRJNA842094 (https://www.ncbi.nlm.nih.gov/bioproject/PRJNA842094) [126]. The genomic data for *L. migratoria* was downloaded in the SRA under accession number SRR764584 (https://www.ncbi.nlm.nih.gov/sra/SRX245287) [127]. Additionally, the TE consensus sequence, transcriptome assembly, and annotation data have been deposited in the figshare database (10.6084/m9.figshare.21256878) [128].

## Abbreviations

TEs: Transposable elements
LTRs: Long terminal repeats
LINEs: Long interspersed nuclear elements
*L. migratoria*: *Locusta migratoria manilensis*
*A. rhodopa*: *Angaracris rhodopa*
WGD: Whole-genome duplication
K2P: Kimura 2-parameter distance
RT: Reverse transcriptase
RH: Ribonuclease H
INT: Integrase
GAG: Group-specific antigen
PROT: Protease
Ty1: Ty1_copia
Ty3: Ty3_gypsy
rRNA: Ribosomal RNA
FCM: Flow cytometry
