# Supplementary material for: Transposable element expansion and low-level piRNA silencing in grasshoppers may cause genome gigantism
Source: BMC Biol. 2022 Oct 28;20:243. doi: 10.1186/s12915-022-01441-w (PMC9615261; doi:10.1186/s12915-022-01441-w)
Supplement: Supplementary file 1 — Additional file 1: Fig. S1. Flow cytometry estimation of the genome size for A. rhodopa female and male. Fig. S2. TE subclass landscapes of two species. Fig. S3. Repeat profiles of the remaining 31 shared TEs between two species. Fig. S4. Low-level piRNA silencing in A.rhodopa ovary. Fig. S5. Correlation analysis of K2P distance of TE with TE abundance and piRNA abundance. [file 12915_2022_1441_MOESM1_ESM.pdf]

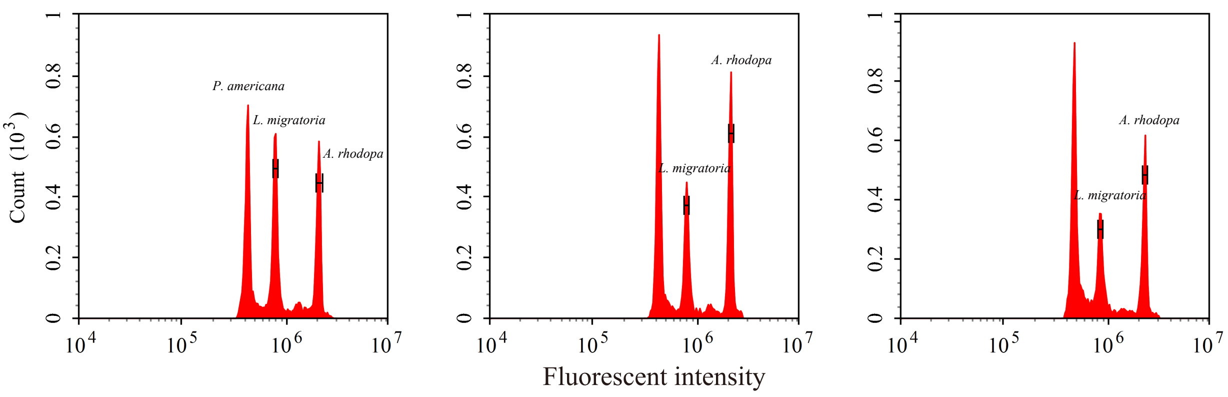


| | Mean | CV | C-value |
|---|---|---|---|
| *Locusta migratoria manilensis* ♂ | 802091 | 2.68% | 6.20 |
| *Angaracis rhodopa* ♀ | 2098539 | 2.69% | 16.221 |

| | Mean | CV | C-value |
|---|---|---|---|
| *Locusta migratoria manilensis* ♂ | 811816 | 2.74% | 6.20 |
| *Angaracis rhodopa* ♀ | 2129988 | 2.66% | 16.267 |

| | Mean | CV | C-value |
|---|---|---|---|
| *Locusta migratoria manilensis* ♂ | 864550 | 2.76% | 6.20 |
| *Angaracis rhodopa* ♀ | 2314528 | 2.42% | 16.598 |

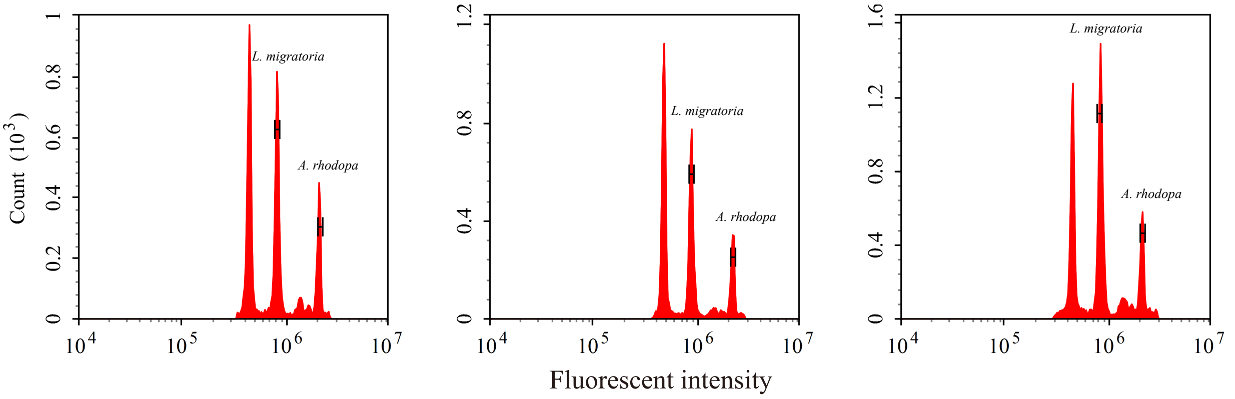


| | Mean | CV | C-value |
|---|---|---|---|
| *Locusta migratoria manilensis* ♂ | 835664 | 2.82% | 6.20 |
| *Angaracis rhodopa* ♂ | 2113545 | 2.56% | 15.681 |

| | Mean | CV | C-value |
|---|---|---|---|
| *Locusta migratoria manilensis* ♂ | 880468 | 2.66% | 6.20 |
| *Angaracis rhodopa* ♂ | 2213249 | 2.54% | 15.585 |

| | Mean | CV | C-value |
|---|---|---|---|
| *Locusta migratoria manilensis* ♂ | 846460 | 2.69% | 6.20 |
| *Angaracis rhodopa* ♂ | 2125756 | 2.56% | 15.570 |

Summary table

| Species | Sex | C-value(pg) | Gnome Size(Gb) |
|---|---|---|---|
| *Angaracis rhodopa* | Female | 16.36 | 16.00204 |
| | Male | 15.61 | 15.26854 |

**Fig. S1 Flow cytometry estimation of the genome size for *A. rhodopa* female and male.** *L. migratoria* ♂（1C=6.20pg）was used as an internal standard.

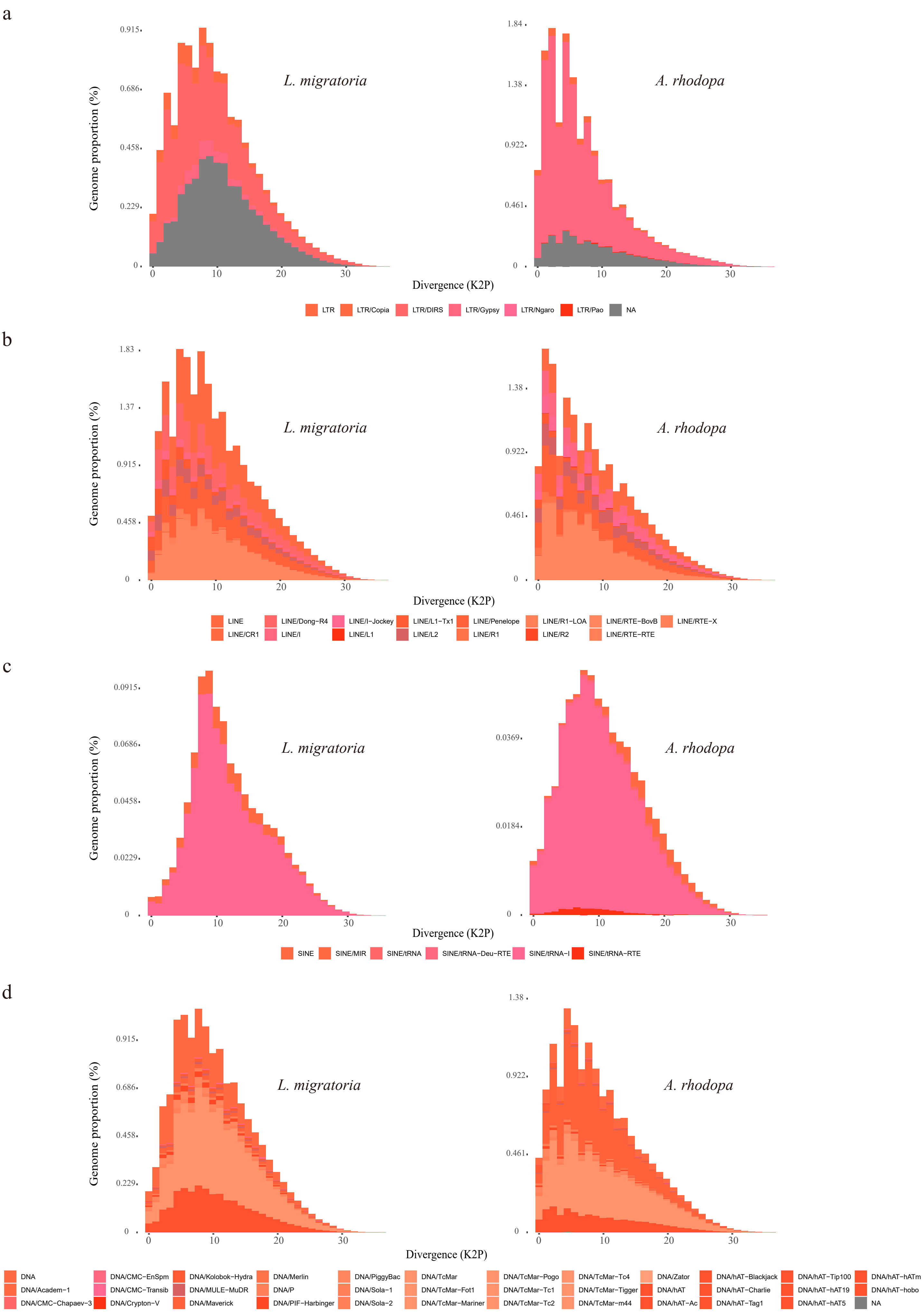


**Fig. S2 TE subclass landscapes of two species**. **a** LTR. **b** LINE. **c** SINE. **d** DNA transposons.

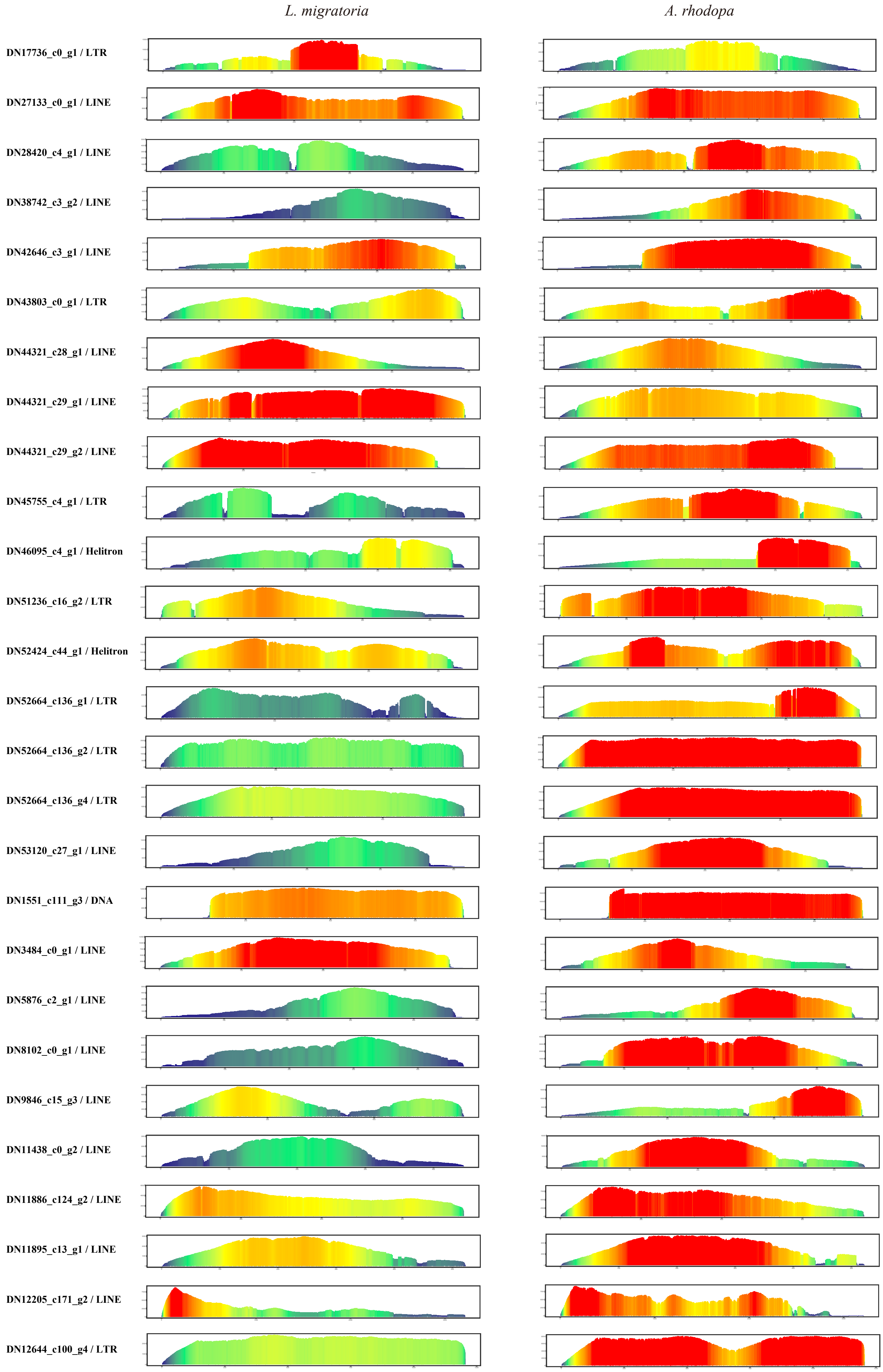
*L. migratoria*
*A. rhodopa*
DN17736_c0_g1 / LTR
DN27133_c0_g1 / LINE
DN28420_c4_g1 / LINE
DN38742_c3_g2 / LINE
DN42646_c3_g1 / LINE
DN43803_c0_g1 / LTR
DN44321_c28_g1 / LINE
DN44321_c29_g1 / LINE
DN44321_c29_g2 / LINE
DN45755_c4_g1 / LTR
DN46095_c4_g1 / Helitron
DN51236_c16_g2 / LTR
DN52424_c44_g1 / Helitron
DN52664_c136_g1 / LTR
DN52664_c136_g2 / LTR
DN52664_c136_g4 / LTR
DN53120_c27_g1 / LINE
DN1551_c111_g3 / DNA
DN3484_c0_g1 / LINE
DN5876_c2_g1 / LINE
DN8102_c0_g1 / LINE
DN9846_c15_g3 / LINE
DN11438_c0_g2 / LINE
DN11886_c124_g2 / LINE
DN11895_c13_g1 / LINE
DN12205_c171_g2 / LINE
DN12644_c100_g4 / LTR

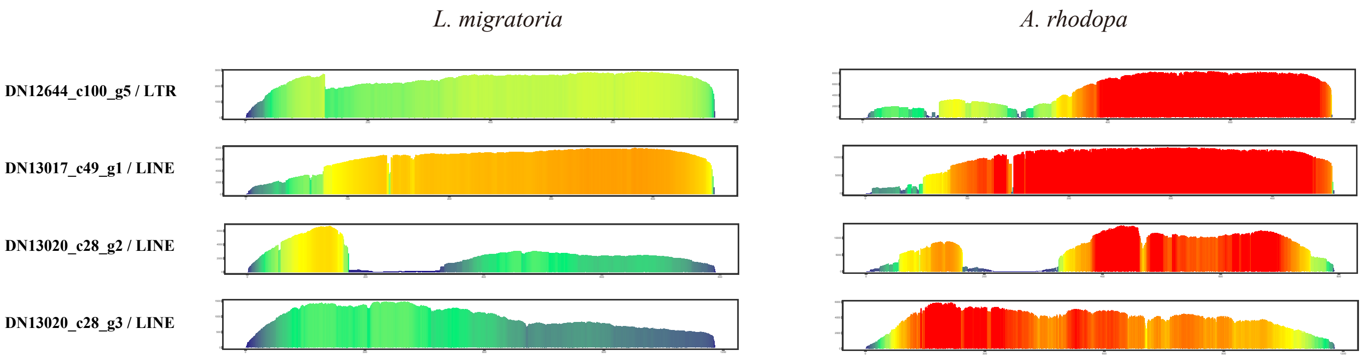


**Fig. S3 Repeat profiles of the remaining 31 shared TEs between two species.** The x-axis represents the loci of the consensus sequence, and the y-axis is the depth of coverage for each position.

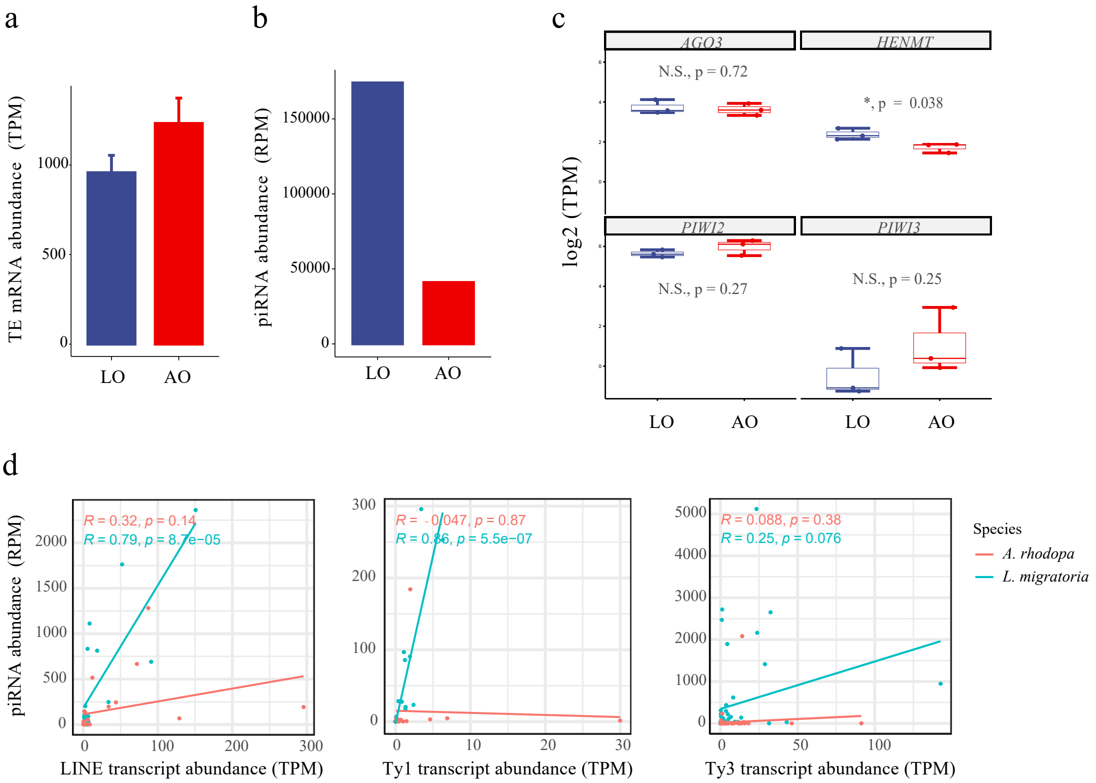


**Fig. S4 Low-level piRNA silencing in *A.rhodopa* ovary**. **a** Total abundance of TE transcripts in the ovary. **b** Total abundance of TE-derived piRNA in the ovary. **c** Analysis of differential expression of four genes in piRNA pathway. The y-axis represents log2 of transcript abundance (TPM). **e** Linear fitting of piRNA abundance and transcript abundance of TEs. Abbrevations are defined as: LO, *L. migratoria* ovary; AO, *A. rhodopa* ovary. Statistical significance is represented as * $p<0.05$; ** $p<0.01$; *** $p<0.001$; N.S. $p>0.05$ (method = T-test; r: Pearson correlation coefficient).

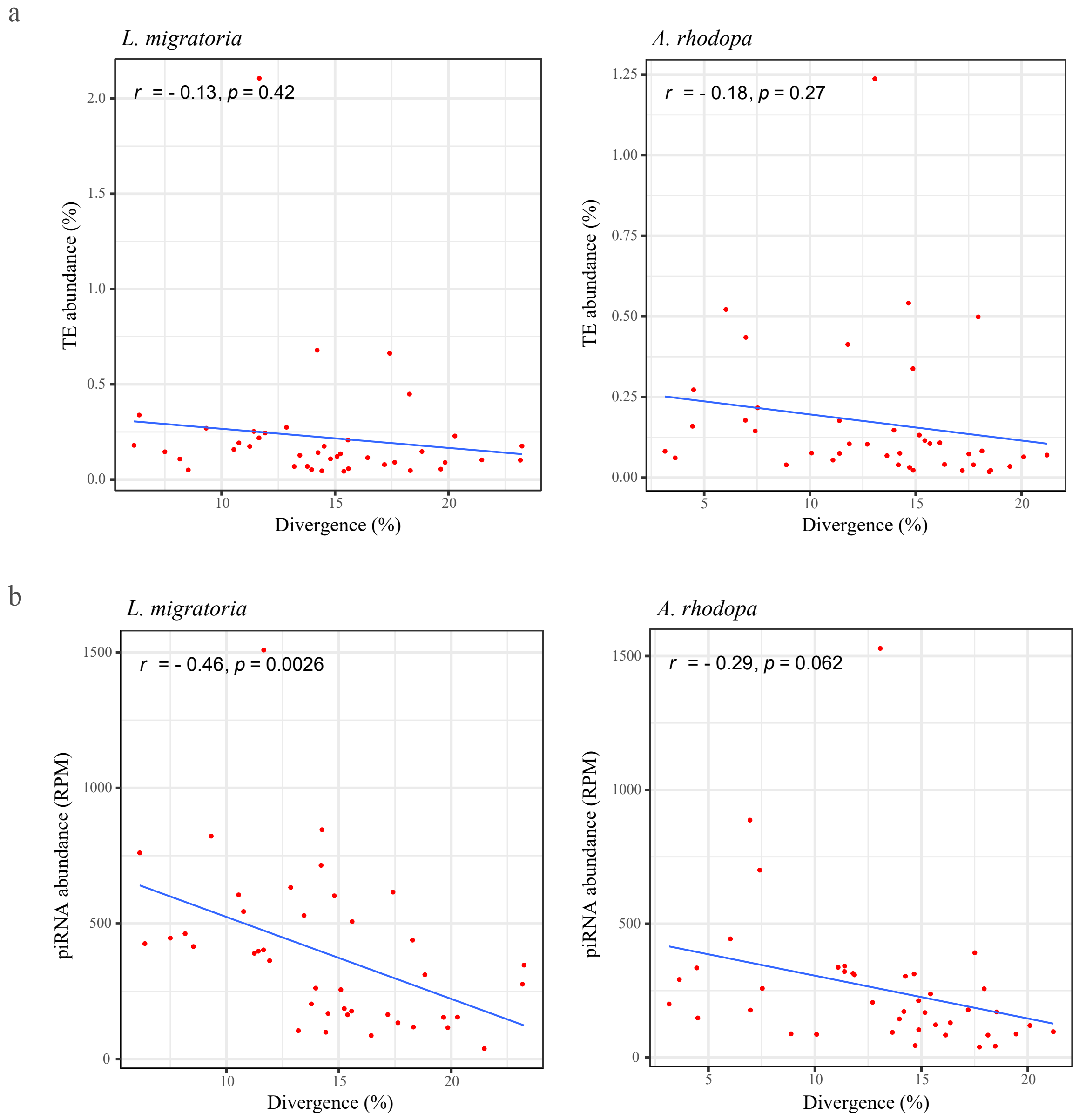


**Fig. S5 Correlation analysis of K2P distance of TE with TE abundance and piRNA abundance.** **a** Correlation analysis of K2P distance and abundance of TEs. The x-axis shows the level of divergence (Kimura 2-parameter distance) between each identified TE copy. The y-axis represents the abundance of TE (genome proportion %). **b** Correlation analysis between the K2P distance of TE and the abundance of piRNA. r: Pearson correlation coefficient.
